# Development and validation of the polycystic ovary syndrome-related complaint severity scale

**DOI:** 10.1590/1806-9282.20250981

**Published:** 2026-03-30

**Authors:** Özlem Kaplan, Mürüvvet Başer, Nurseli Soylu Erener, Ali Kaplan, Busra Emir, Gökhan Açmaz

**Affiliations:** 1 Erciyes University, Faculty of Health Sciences, Department of Obstetrics and Gynecology Nursing – Kayseri, Türkiye.; 2 University of Kayseri, İncesu Ayşe and Saffet Arslan Health Services Vocational School, Department of Medical Services and Techniques – Kayseri, Türkiye.; 3 University of İzmir Kâtip Çelebi, Faculty of Medicine, Department of Biostatistics – İzmir, Türkiye.; 4 Erciyes University, Faculty of Medicine, Department of Obstetrics and Gynecology – Kayseri, Türkiye.

**Keywords:** Polycystic ovary syndrome, Surveys and questionnaires, Complaints

## Abstract

**OBJECTIVE:**

The aim of this study was to develop and validate the Severity of the Complaints Related to Polycystic Ovary Syndrome Scale.

**METHODS:**

This methodological study employed exploratory factor analysis and confirmatory factor analysis. Internal consistency was assessed through item–total correlations and the test–retest method.

**RESULTS:**

Exploratory factor analysis revealed a four-factor structure explaining 57.416% of the variance. Confirmatory factor analysis results confirmed a good model fit (χ^2^/df=3.094, RMSEA=0.065, CFI=0.913, GFI=0.910). The overall Cronbach’s alpha coefficient was 0.897. In the test–retest reliability analyses, significant correlations were found between all items and factor scores across the two administrations (p<0.001).

**CONCLUSION:**

The scale is a valid and reliable measure for assessing the severity of complaints related to Polycystic Ovary Syndrome Scale.

## INTRODUCTION

Polycystic ovary syndrome (PCOS) is a multifactorial endocrine disorder affecting women of reproductive age^1^. PCOS can be seen in approximately 9.2% of women of reproductive age^2,3^. Women diagnosed with PCOS have an increased risk of insulin resistance, type 2 diabetes, cardiovascular diseases, and infertility, as well as depression and anxiety^3,4,5^. Symptoms such as dyslipidemia, hypertension, hyperinsulinemia, sugar cravings, frequent urination, delayed healing of wounds, fatigue, blurred vision, tingling, mood changes, obesity, skin fat, and spotting are commonly seen in these patients. In addition, complaints such as pelvic pain, fever, nausea, vomiting, urinary system problems, irritable bowel syndrome, constipation, sleep apnea, and deterioration in body image are also frequently experienced^6,7^.

PCOS is a multifaceted condition that negatively impacts women’s quality of life and can lead to serious short- and longterm health issues. Currently, no reliable and valid tool in the literature measures the physiological, psychological, and social complaints specific to PCOS. This study aims to fill this critical gap by developing a new measurement instrument to quantitatively assess the severity of PCOS-related complaints.

## METHODS

### Study design and procedure

This prospective, cross-sectional, and descriptive study was conducted in Türkiye between May and August 2024, involving 496 women diagnosed with PCOS.

### Conceptual framework

The conceptual framework of the scale development study focused on determining the severity of complaints experienced by patients due to PCOS. It creates a structure for determining women’s physiological, psychological, and social complaints that may lead to short- and long-term health problems due to PCOS. In line with this conceptual framework, a literature review was conducted on a large number of articles, including systematic reviews, meta-analyses, guidelines, and qualitative studies, in which PCOS symptoms and complaints were determined^3,4,5,7,8^. In addition, the development process benefited from the experiences, observations, and insights gained through patient care practices by physicians and researchers, ensuring that the scale items were grounded in real-world clinical contexts. The qualitative findings obtained from these interviews were analyzed using inductive content analysis^9^, resulting in 32 preliminary items for the initial version of the scale.

### Content analysis

Content validity was assessed using the Lawshe technique^10^. Expert evaluations were conducted in two stages to review the 32 preliminary items. Based on feedback, three items were removed and others revised. For 20 experts, a CVR≥0.42 was acceptable at α=0.05^11^. Item CVRs ranged from 0.85 to 1.0, with an overall CVI of 0.96. Language revisions by three experts finalized the scale as a 29-item scale, and responses were rated on a five-point Likert scale ranging from 1 (never) to 5 (always).

### Participants

Women diagnosed with PCOS by a physician were included in the study. Based on the recommendation of having at least 10 participants per item for a 29-item scale, a minimum sample size of 290 was required^12^, and a final sample of 496 was confirmed adequate via the KMO test.

**Figure 1:**
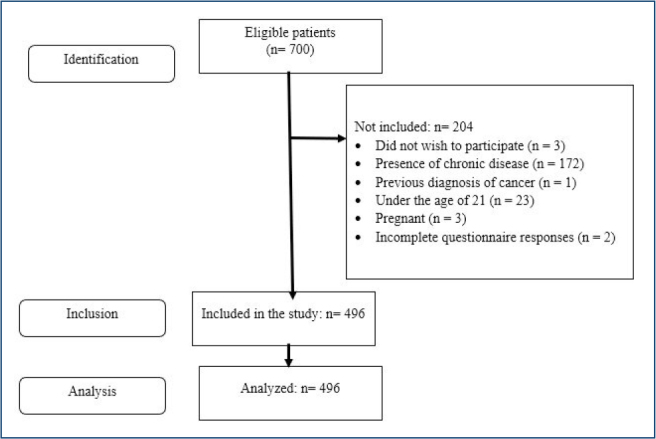
Study flow diagram.

A total of 700 women were contacted, but 204 were excluded (Figure 1). Inclusion criteria were women aged 21 or older, diagnosed with PCOS by a physician, who gave informed consent online, and completed all forms. Exclusion criteria included pregnancy, menopause, chronic diseases (e.g., non-classical 21-hydroxylase deficiency, type 1 diabetes mellitus, acute or chronic renal failure, thyroid dysfunction, Cushing’s syndrome, hyperprolactinemia), and history of cancer.

### Data collection

Data were collected in Türkiye between May and August 2024 from women diagnosed with PCOS. The questionnaire, prepared via Google Forms, was sent by text message. To prevent duplicate entries, responses were restricted to one per IP address. For reliability assessment, a test–retest procedure was conducted with 50 participants, and the second administration of the scale was performed four weeks after the first one. Data were collected through two instruments: an 18-item Descriptive Characteristics Form and the 29-item draft of the PCOS-Related Complaints Scale.

### Data analysis

Statistical analyses were conducted using IBM SPSS Statistics v.25 (IBM Corp., Armonk, NY, USA). Descriptive statistics were presented as frequency (n), percentage (%), mean ± standard deviation, minimum–maximum, or median (IQR). The Shapiro-Wilk test and Q-Q plots were used to assess the normality of numerical variables. Content validity ratios were calculated. For the PCOS-Related Complaints Scale, construct validity, internal consistency reliability, test–retest reliability, and item analysis were performed. Exploratory factor analysis (EFA) and confirmatory factor analysis (CFA) were used to assess construct validity. It was assumed that the four latent factors in the model (Factor 1, Factor 2, Factor 3, and Factor 4) could be conceptually related to each other, and therefore, correlations among them were allowed. Before EFA, Bartlett’s test of sphericity and the Kaiser-Meyer-Olkin (KMO) test were conducted to determine data suitability and sample adequacy^13^. EFA was performed using the principal components method with varimax rotation, resulting in a four-factor structure. Cronbach’s alpha coefficient was calculated for internal consistency reliability. Spearman correlation analysis was used to evaluate the relationship between total and subscale scores. CFA was performed using IBM AMOS v.23 to test whether the factor structure identified in the EFA fit the observed data. Model fit was assessed using fit indices including χ^2^/df, SRMR, RMSEA, CFI, GFI, AGFI, NFI, and NNFI. A significance level of p<0.05 was considered statistically significant^14^.

### Ethical considerations

This study was performed following the Helsinki Declaration and has been approved by the Kayseri University’s Ethics Committee University’s Ethics Committee (approval date/number: 26.03.2024/94141). All participants were provided with detailed information about the study’s purpose and scope. Informed consent was obtained through an online consent checkbox labeled “I Agree” on the survey form.

## RESULTS

The mean age of the women was 27.20±4.34 (min: 20–max: 45) years, and the mean body mass index was 27.73±6.25 kg/m^2^. On average, they had been married for 4.54±4.55 years, and 52.4% were single. The mean diagnosis of PCOS was 83.83±66.42 months, and 62.1% of the participants had not received any medical treatment for this condition before. The mean duration of treatment for PCOS was 13.32±26.68 months. In addition, the mean menstrual cycle duration was 55.12±62.34 days, and the mean menstrual bleeding duration was 6.06±3.72 days. It was also determined that the participants experienced their last menstrual bleeding an average of 31.62±55.99 days prior.

A corrected item–total correlation above 0.40 is recommended in the literature^15^. Items 7–14 and 17, which fell below this threshold, were removed step by step, and scale statistics were recalculated. The final analysis was conducted on the remaining 20 items (Table 1).

**Table 1:** Reliability coefficients of the scale (n=496).

	First step				Last step			
	Scale mean if item deleted	Scale variance if item deleted	Corrected item–total correlation	Cronbach’s alpha if item deleted	Scale mean if item deleted	Scale variance if item deleted	Corrected item–total correlation	Cronbach’s alpha if item deleted
Item-1	92.4758	276.969	0.448	0.892	64.1774	166.954	0.458	0.894
Item-2	92.0585	275.708	0.418	0.892	63.7601	165.476	0.444	0.894
Item-3	92.9234	273.138	0.454	0.891	64.6250	165.415	0.412	0.895
Item-4	92.9536	270.198	0.500	0.891	64.6552	163.265	0.455	0.894
Item-5	92.1754	274.707	0.519	0.891	63.8770	166.011	0.497	0.893
Item-6	92.2500	275.638	0.468	0.891	63.9516	167.840	0.403	0.895
Item-7	92.6956	275.735	**0.387**	0.893	–	–	–	–
Item-8	92.9758	277.660	**0.346**	0.894	–	–	–	–
Item-9	94.5484	281.283	**0.309**	0.894	–	–	–	–
Item-10	93.5444	281.550	**0.246**	0.896	–	–	–	–
Item-11	92.7944	277.602	**0.355**	0.893	–	–	–	–
Item-12	92.7984	278.270	**0.342**	0.894	–	–	–	–
Item-13	92.7036	278.629	**0.300**	0.895	–	–	–	–
Item-14	92.8488	274.533	**0.364**	0.894	–	–	–	–
Item-15	92.7056	274.653	0.417	0.892	64.4073	165.854	0.400	0.896
Item-16	94.2823	272.607	0.467	0.891	65.9839	163.685	0.469	0.894
Item-17	92.6431	278.347	**0.258**	0.896	–	–	–	–
Item-18	92.2319	273.855	0.609	0.889	63.9335	164.854	0.609	0.890
Item-19	92.3891	271.111	0.571	0.889	64.0907	162.935	0.561	0.891
Item-20	92.5786	273.792	0.576	0.890	64.2802	163.952	0.612	0.890
Item-21	92.4839	274.703	0.505	0.891	64.1855	164.729	0.534	0.892
Item-22	92.1310	270.280	0.571	0.889	63.8327	160.673	0.621	0.889
Item-23	93.4536	264.357	0.610	0.888	65.1552	156.447	0.640	0.888
Item-24	93.5988	265.748	0.596	0.888	65.3004	157.136	0.639	0.888
Item-25	93.8508	268.903	0.496	0.891	65.5524	159.185	0.547	0.891
Item-26	93.1472	264.530	0.602	0.888	64.8488	156.504	0.634	0.888
Item-27	93.6653	269.985	0.461	0.891	65.3669	160.354	0.500	0.893
Item-28	92.1694	275.559	0.558	0.890	63.8710	165.414	0.593	0.891
Item-29	92.6815	270.044	0.510	0.890	64.3831	162.245	0.494	0.893

Values in bold represent findings below 0.40.

**Figure 2:**
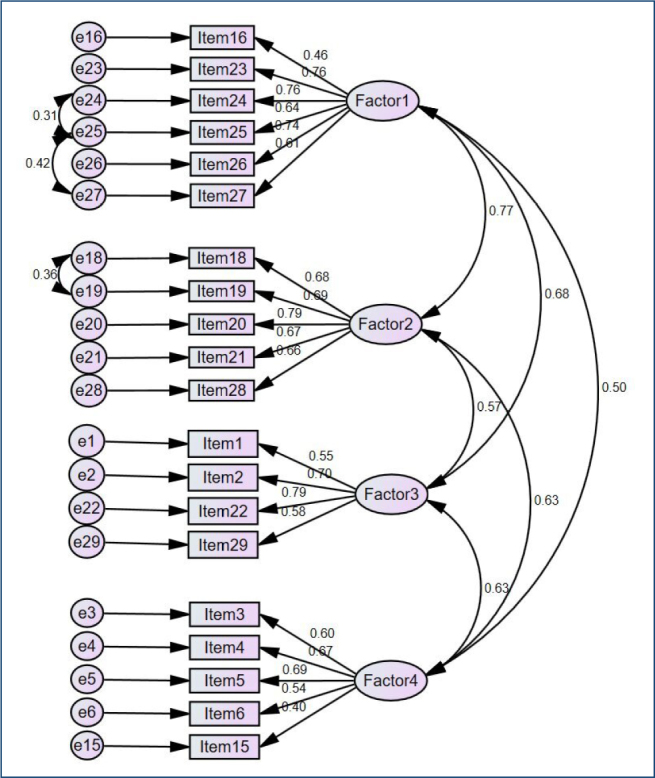
Path diagram.

ANOVA with Tukey’s test for non-additivity revealed that the scale has a summable structure (F=78.542, p<0.001). The KMO value was 0.904, and Bartlett’s test of sphericity was significant (χ^2^=4002.377, p<0.001), confirming the suitability of the data for factor analysis. Principal component analysis with varimax rotation revealed a four-factor structure explaining 57.416% of the total variance. These factors were identified as systemic and social impact complaints (items 16, 23–27), emotional complaints (items 18–21, 28), complaints related to physical appearance (items 1, 2, 22, 29), and physiological complaints (items 3–6, 15). Cronbach’s alpha values for the subscales ranged from 0.704 to 0.842, indicating good internal consistency. CFA confirmed the four-factor model, and fit indices demonstrated an acceptable model fit according to standard criteria (Table 2 and Figure 2)^16^.

**Table 2:** Exploratory and confirmatory factor analysis (n=496).

Items		Factor 1	Factor 2	Factor 3	Factor 4	% of variance explained	Cronbach’s alpha
Item-25	I hesitate to have romantic relationships.	0.849	0.094	0.075	0.123	35.053	0.842
Item-27	I hesitate to have sexual intimacy.	0.816	0.113	0.072	0.041		
Item-24	I avoid going into social environments.	0.754	0.223	0.146	0.218		
Item-23	I don’t really feel like a woman anymore.	0.603	0.314	0.070	0.390		
Item-26	I think that my complaints affect my family life negatively.	0.552	0.467	0.130	0.192		
Item-16	There are times when my breathing stops (apnea), I feel choked, or I wake up with a feeling of suffocation.	0.358	0.236	0.306	0.124		
Item-19	I experience sudden changes in emotion such as laughing, crying, or getting angry.	0.047	0.821	0.221	0.150	9.577	0.833
Item-20	I feel unhappy.	0.333	0.772	0.074	0.122		
Item-18	I feel tense and nervous.	0.140	0.748	0.181	0.253		
Item-21	I have difficulty concentrating.	0.311	0.579	0.278	-0.021		
Item-28	I feel tired during the day.	0.289	0.471	0.467	0.060		
Item-2	I gain weight quickly and have difficulty losing weight.	0.074	0.031	0.159	0.854	7.331	0.746
Item-22	I am not satisfied with my physical appearance.	0.317	0.249	0.151	0.694		
Item-29	I have a swelling (edema) problem in different parts of my body such as hands, face, and legs.	0.163	0.188	0.204	0.610		
Item-1	I can’t control my appetite.	0.117	0.060	0.386	0.534		
Item-4	I experience symptoms such as sudden hunger, trembling hands and feet, cold sweating.	0.073	0.134	0.787	0.061	5.455	0.704
Item-3	I am experiencing thirst and dry mouth.	0.065	0.060	0.685	0.160		
Item-5	I feel sleepy and heavy after eating.	0.065	0.258	0.670	0.150		
Item-6	I have gastrointestinal problems such as indigestion, gas, and bloating.	0.041	0.105	0.582	0.230		
Item-15	I have a sleep disorder.	0.199	0.169	0.367	0.183		
Total						57.416	0.897
Confirmatory factor analysis results							
Goodness-of-fit indices		χ^2^/df	RMSEA	SRMR	CFI	GFI	NNFI
Reference value		<3 (<0.05)	≤0.08	≤0.10	≥0.90	≥0.85	≥0.90
Model		3.094 (<0.001)	0.065	0.060	0.913	0.910	0.883

Factor 1: Systemic and social impact complaints, Factor 2: Emotional complaints, Factor 3: Complaints related to physical appearance, Factor 4: Physiological complaints. Subtraction method: principal component analysis, Rotation method: varimax, rotation was combined after seven iterations, χ^2^/df: Chi-squared/degrees of freedom, RMSEA: Root mean square error of approximation, SRMR: standardized root mean squared residual, CFI: comparative fit index, GFI: goodness-of-fit. NNFI: Non-Normed fit index.

As a result of the CFA, it was observed that the four latent variables in the model (Factor 1: Systemic and Social Impact Complaints, Factor 2: Emotional Complaints, Factor 3: Complaints Related to Physical Appearance, and Factor 4: Physiological Complaints) were positively and significantly correlated with each other. The correlation coefficients among the factors ranged from 0.50 to 0.77. The highest correlation between Factor 1 and Factor 2 (r=0.77) indicated a strong conceptual relationship between these two dimensions. The correlations between Factor 1 and Factor 4, Factor 2 and Factor 4, Factor 3 and Factor 4, and Factor 2 and Factor 3 were r=0.50, r=0.63, r=0.63, and r=0.57, respectively. These findings suggest that the factors are not entirely independent; however, the structural distinctiveness of the scale is maintained. Therefore, conceptual overlaps among factors are expected and consistent with the nature of multidimensional scales (Figure 2).

Regarding the relationships between the scale and continuous variables, a weak positive correlation was found between the total score and body mass index (BMI) (r=0.324; p<0.001), while a weak negative correlation was found between the total score and menstrual bleeding duration (r=-0.101; p=0.024). No significant correlations were found between the total score and age, duration of marriage, duration of PCOS diagnosis, duration of PCOS treatment, menstrual cycle length, or the time since the last menstrual period (p>0.05). Among the sub-dimensions, Factor 1 was weakly and positively correlated with BMI (r=0.254; p<0.001) and duration of PCOS diagnosis (r=0.115; p=0.010). Factor 2 was weakly and negatively correlated with age (r=-0.135; p=0.003). Factor 3 was weakly and positively correlated with BMI (r=0.522; p<0.001). Factor 4 was weakly and positively correlated with BMI (rho=0.193; p<0.001) and negatively correlated with age (rho=-0.104; p=0.020) and menstrual bleeding duration (rho=-0.200; p=0.001). No other significant correlations were detected between the sub-dimensions and continuous variables (p>0.05) (Table 3).

**Table 3:** Examination of the relationship between the total scale score, subscales, and other variable (n=496).

	Correlation coefficient p-value	Total	Factor 1	Factor 2	Factor 3	Factor 4	BMI	Age	Time of married	PCOS month	PCOS treatment month	Menstrual cycle duration day	Menstrual bleeding duration day	How many days ago was your last period
Total	Rho	1.000												
	p-value													
Factor 1	Rho	0.846	1.000											
	p-value	**<0.001**												
Factor 2	Rho	0.792	0.585	1.000										
	p-value	**<0.001**	**<0.001**											
Factor 3	Rho	0.755	0.523	0.467	1.000									
	p-value	**<0.001**	**<0.001**	**<0.001**										
Factor 4	Rho	0.716	0.386	0.497	0.510	1.000								
	p-value	**<0.001**	**<0.001**	**<0.001**	**<0.001**									
BMI	Rho	0.324	0.254	0.084	0.522	0.193	1.000							
	p-value	**<0.001**	**<0.001**	0.061	**<0.001**	**<0.001**	.							
Age	Rho	-0.080	-0.015	-0.135	-0.011	-0.104	0.106	1.000						
	p-value	0.076	0.737	**0.003**	0.805	**0.02**	**0.019**							
Time of marriage	Rho	0.047	0.120	-0.001	0.067	-0.034	0.167	0.695	1.000					
	p-value	0.468	0.062	0.991	0.298	0.600	**0.009**	**<0.001**						
PCOS month	Rho	0.06	0.115	-0.017	0.044	0.002	0.068	0.384	0.274	1.000				
	p-value	0.182	**0.010**	0.704	0.327	0.970	0.130	**<0.001**	**<0.001**	.				
PCOS treatment month	Rho	0.075	0.067	0.034	0.079	0.068	-0.017	-0.091	-0.211	0.116	1.000			
	p-value	0.109	0.156	0.475	0.094	0.152	0.715	0.053	**0.002**	**0.014**				
Menstrual cycle duration, days	Rho	0.071	0.040	0.086	0.027	0.081	0.050	-0.067	-0.045	0.077	-0.149	1.000		
	p-value	0.113	0.378	0.055	0.553	0.073	0.270	0.136	0.489	0.088	**0.001**	.		
Menstrual bleeding duration day	Rho	-0.101	-0.049	-0.047	-0.082	-0.148	-0.024	0.021	0.163	0.027	-0.065	0.133	1.000	
	p-value	**0.024**	0.273	0.295	0.069	**0.001**	0.601	0.64	**0.011**	0.542	0.168	**0.003**	.	
How many days ago was your last period	Rho	0.085	0.100	0.071	0.054	0.029	0.078	-0.033	-0.049	0.061	-0.036	0.425	0.026	1.000
	p-value	0.059	**0.025**	0.113	0.231	0.517	0.081	0.460	0.450	0.178	0.449	**<0.001**	0.562	.

Rho: Spearman’s correlation coefficient. Values in bold (<0.05) are considered statistically significant. BMI: body mass index; PCOS: Polycystic Ovary Syndrome Scale.

A small but statistically significant difference was observed in Factor 1 between the test and retest. The first measurement mean rank score was higher than the second measurement mean rank score (p=0.012). However, this reflected only a systematic yet minimal mean shift that did not impair reliability (ICC=0.987). For Factor 2, Factor 3, Factor 4, and total scores, similarity was found between the first and second scores obtained from the scale (p>0.05). There was a high level of agreement between the first and second measurements, as indicated by the ICC values. Additionally, the Cronbach’s alpha value of the scale was found to be 0.856 in the analysis performed using the test–retest method. According to these findings, the scale is reliable (Table 4).

**Table 4:** Test–retest results (n=50).

	First measurement Mean±SD Median (IQR)	Second measurement Mean±SD Median (IQR)	Test statistics	p-value	ICC	p-value
Factor 1	16.42±5.82 16 (8)	15.94±6.14 14.5 (9)	3.136	0.002	0.987 (0.977–0.992)	**<0.001**
Factor 2	19.64±2.73 19 (4)	19.72±2.99 20 (4.25)	0.703	0.482	0.936 (0.889–0.963)	**<0.001**
Factor 3	15.78±3.61 17 (6)	15.78±3.61 17 (6)	1.659	0.097	0.971 (0.949–0.983)	**<0.001**
Factor 4	18.74±3.47 19 (5)	18.90±3.63 19 (4)	1.171	0.242	0.959 (0.929–0.977)	**<0.001**
Total	70.38±11.47 69 (16.75)	70.34±12.47 69.5 (16.25)	0.212	0.832	0.982 (0.968–0.990)	**<0.001**

ICC: Intraclass correlation coefficient; SD: standard deviation, Wilcoxon signed ranks test. Values in bold (<0.05) are considered statistically significant.

## DISCUSSION

The PCOS-related complaint severity scale was developed to fill the gap in measuring the specific intensity of PCOS-related symptoms. Unlike quality-of-life tools^17^, this scale focuses on symptom severity and supports multidisciplinary care. It consists of four factors and 20 items. Content validity refers to how well scale items represent the targeted construct and is typically evaluated by experts^18^. In this study, the Lawshe technique was applied, which recommends involving 5–40 experts^19^. Feedback from 20 experts indicated that all items had CVR values ≥0.85 (threshold=0.42)^17^, and the overall CVI was 0.96, confirming strong content validity.

Item analysis evaluates how well each item contributes to the overall reliability and internal structure of a scale^20^. A corrected item–total correlation above 0.40 is recommended in the literature^21^. In this study, nine items below this threshold were removed. The remaining 20 items had correlations ranging from 0.400 to 0.640, indicating that they are distinctive and consistently measure the intended construct^12^. Factor analysis is used to identify the underlying structure of observed variables by grouping-related items into core factors^22^. To assess sample adequacy, the KMO and Bartlett’s tests were applied. A KMO value above 0.80 is acceptable; in this study, it was 0.904^23^, and Bartlett’s test was statistically significant (p<0.001). These results indicate that the sample was sufficient and appropriate for factor analysis.

In factor analysis, axis rotation clarifies factor structure, and a total explained variance of at least 30% is recommended for multidimensional scales^12^. In this study, four factors emerged from 20 items, explaining 57.416% of the total variance (35.053, 9.577, 7.331, and 5.455%, respectively). Out of which, 40–60% of the variance is considered sufficient in scale development^24^, indicating a strong internal structure. The scale also demonstrated high reliability, with a Cronbach’s alpha of 0.897.

As shown in Table 2, factor analysis grouped the items into four dimensions: systemic and social impact complaints (6 items), emotional complaints (5 items), complaints about physical appearance (4 items), and physiological complaints (5 items). Cronbach’s alpha values for the factors were 0.842, 0.833, 0.746, and 0.704, respectively. Since values above 0.70 indicate acceptable reliability^16^, the scale and sub-dimensions demonstrate strong internal consistency and reliability. CFA tests whether the proposed factor structure fits the data and confirms the sub-dimensions identified during construct validity assessment. Acceptable fit is indicated by χ^2^/df between 2 and 3, RMSEA≤0.08, p<0.05, SRMR<0.10, and CFI, GFI, NNFI≥0.90^16^. The analysis results showed that the model demonstrated an acceptable fit to the data (χ^2^/df=3.094, p<0.001, RMSEA=0.065, SRMR=0.060, CFI=0.913, GFI=0.910, NNFI=0.883). Although the NNFI value was slightly below the ideal threshold, it was close to the acceptable limit, and all other indices indicated a satisfactory model fit, supporting the adequacy of the four-factor structure.

The test–retest method, commonly used to assess reliability, expects a correlation above 0.70 between repeated measurements^20,25^. In this study, test–retest correlations were 0.987 (Factor 1), 0.936 (Factor 2), 0.971 (Factor 3), 0.959 (Factor 4), and 0.982 for the total score, all statistically significant (p<0.001). These results confirm the scale’s high reliability. The severity of complaints related to PCOS Scale is a valid and reliable 20-item tool with four sub-dimensions, scored on a 5-point Likert scale. The total score ranges from 20 to 100, with higher scores indicating greater severity of PCOS-related complaints.

Convergent validity refers to the extent to which a newly developed instrument correlates positively with other measures that assess similar or related constructs, thereby demonstrating that it measures the intended concept. In the literature, convergent validity is typically evaluated by examining correlations between the new scale and existing validated instruments. However, in the absence of a gold-standard or comparable tool measuring the same construct, it is also acceptable to assess convergent validity through correlations with relevant clinical or psychological variables that are theoretically expected to be associated with the construct^16,22^. In this study, convergent validity was examined through correlation analyses between the scale scores and continuous variables, including age, BMI, menstrual characteristics, and duration of PCOS diagnosis and treatment. These associations provided empirical support consistent with theoretical expectations regarding PCOS-related symptom severity. Although this approach offers preliminary evidence of construct validity, future research could further evaluate convergent validity by comparing the scale scores with other standardized psychological or clinical measures, such as instruments assessing depression, anxiety, body image, or hyperandrogenism.

### Limitations

This study has some limitations. Although the scale development process included interviews with patients conducted by physicians and experienced researchers, more extensive qualitative studies could further enrich the item pool and enhance content validity. The sample primarily consisted of Turkish women; therefore, the findings may not be generalizable to other cultures. The criterion validity of the scale was not evaluated against a gold-standard measure. However, convergent validity was partially examined through correlation analyses with continuous variables within this study. Future studies are recommended to further assess convergent validity by comparing the scale scores with other established psychological or clinical instruments.

## CONCLUSION

This study introduced a valid and reliable scale to assess the severity of PCOS-related complaints. It provides a practical tool for clinical and research settings, facilitating symptom evaluation, individualized care planning, and monitoring interventions to improve patient outcomes and satisfaction.

## Data Availability

The datasets generated and/or analyzed during the current study are available from the corresponding author upon reasonable request.
